# Selective micro-structural integrity impairment of the isthmus subregion of the corpus callosum in alcohol-dependent males

**DOI:** 10.1186/s12888-019-2079-6

**Published:** 2019-03-25

**Authors:** Yajun Wang, Xiaohu Li, Cun Zhang, Haibao Wang, Zipeng Li, Jiajia Zhu, Yongqiang Yu

**Affiliations:** Department of Radiology, The First Affiliated Hospital of Anhui Medical University, Hefei, 230022 Anhui Province China

**Keywords:** Alcohol dependence, Corpus callosum, Diffusion tensor imaging, Tractography, Subregion

## Abstract

**Background:**

Previous studies have provided evidence that alcohol-dependent patients have abnormality in corpus callosum (CC); however, it is unclear whether micro-structural integrity of the CC subregions is differentially affected in this disorder.

**Methods:**

In this study, a total of 39 male individuals, including 19 alcohol-dependent patients and 20 age-matched healthy controls, underwent diffusion tensor imaging (DTI). CC was reconstructed by DTI tractography and was divided into seven subregions. Multiple diffusion metrics of each subregion were compared between two groups.

**Results:**

Compared to healthy controls, patients exhibited increased axial diffusivity (*P* = 0.007), radial diffusivity (*P* = 0.009) and mean diffusivity (*P* = 0.005) in the isthmus. In addition, we observed that daily alcohol intake was correlated positively with radial diffusivity and mean diffusivity and negatively with fractional anisotropy, while abstinence time of hospitalization was negatively correlated with mean diffusivity in the patients.

**Conclusion:**

These findings suggest a selective micro-structural integrity impairment of the corpus callosum subregions in alcohol dependence, characterized by axon and myelin alterations in the isthmus.

**Electronic supplementary material:**

The online version of this article (10.1186/s12888-019-2079-6) contains supplementary material, which is available to authorized users.

## Background

Alcohol dependence is a severe psychiatric disorder characterized by a chronic self-regulation failure in regard to alcohol consumption, which results in negative physiological, psychological and societal consequences [1–4]. Behavioral evidence has suggested that alcoholics have deficits in motor function [5, 6] and various cognitive domains including memory, attention, execution and social cognition [7–10]. With the development of magnetic resonance imaging (MRI), previous studies have found widespread brain white matter impairments in addictive disorders [11–22] including alcohol dependence [11–16, 20–22] and these micro-structural integrity abnormalities were usually detected by using diffusion tensor imaging (DTI) [13–16, 20–22]. Among affected white matter tracts, corpus callosum (CC) is the most prominent, with primary abnormal features being reduced volume and disrupted micro-structural integrity [20–27]. Since CC connects bilateral hemispheres and relays sensory, motor and cognitive information between them [28–30], one may speculate that CC impairments may contribute to the clinical features in alcohol-dependent patients.

CC can be divided into several subdivisions based on their anatomical landmarks or the specific brain regions that these subdivisions connect [26, 31]. Investigating changes of CC at the subregional level may further improve our understanding of the CC role in many brain diseases, such as post-traumatic stress disorder [32], bipolar disorder [33], autism [34] and schizophrenia [35]. To our knowledge, one prior tractography-based segmentation study has found that all segments of the CC exhibited lower fractional anisotropy (FA) in alcohol dependence and the segment connecting the bilateral orbifrontal cortices was the most affected [26]. For DTI, several other frequently used metrics, such as axial diffusivity (AD), radial diffusivity (RD) and mean diffusivity (MD), could provide more directionally specific and complementary information [36, 37]. The combination of multiple diffusion metrics may offer important insights into the underlying pathological changes of white matter in alcohol dependence.

In the current study, we aimed to systematically test the micro-structural integrity differences of each CC subregion between alcohol-dependent patients and healthy controls using multiple diffusion metrics derived from DTI data.

## Methods

### Participants

A total of 39 right-handed males (19 alcohol-dependent patients and 20 healthy controls) were included in the present study. Alcohol-dependent patients were recruited from the inpatient department at Hefei Fourth People’s Hospital with a mean age of 38.7 years (range: 21–51, SD: 7.8). Healthy controls were recruited from the local community via advertisements with an average age of 42.9 years (range: 24–55, SD: 11.5). Patient and control groups did not differ in age (*t* = 1.30, *df* = 37, *P* = 0.20). This study was approved by the Medical Research Ethics Committee of The First Affiliated Hospital of Anhui Medical University and was performed in line with the principles of the Declaration of Helsinki. Each participant gave written informed consent prior to all study procedures.

Patients’ diagnoses of alcohol dependence were based on the Structured Clinical Interview for DSM-IV Axis I Disorder, Patient Edition (SCID-P) [38]. Patients with three or more of the following criteria at any time in the same 12-month period were included: (1) tolerance; (2) withdrawal; (3) alcohol use in larger amounts or over a longer period than was intended; (4) an unsuccessful effort or persistent desire to cut down or control alcohol use; (5) a great deal of time being spent in activities necessary to obtain or use alcohol or recover from its effects; (6) important social, occupational or recreational activities reduced or given up; and (7) continuation of use despite a persistent or recurrent physical or psychological problem. All patients were hospitalized when they were firstly diagnosed to have alcohol dependence in the outpatient department. Healthy controls were excluded if they had any current psychiatric axis I disorder or history of an addiction according to the non-patient edition of the SCID (SCID-NP). 17 alcohol-dependent patients had available data of daily alcohol intake (497.1 ± 132.8 ml), dependence duration (14.6 ± 7.2 years) and abstinence time of hospitalization (34.6 ± 23.6 days). The healthy controls either had no drinking use or had a low frequency of drinking use that could not be quantified. None of the individuals reported illicit drug use. The details of demographic and clinical data of the participants are shown in Table 1. Exclusionary criteria for all individuals were significant neurological and medical diagnoses, claustrophobia. All participants were requested to abstain from any alcohol and caffeinated beverages at least 24 h prior to the scans. At the day of scanning, subjects were excluded if they had a positive alcohol breathalyzer or urine drug screen.

**Table 1:** Demographic and clinical characteristics

Characteristics	Alcohol-dependent patients	Healthy controls	Statistics	*P* value
Number of subjects	19	20		
Age (years)	38.7 ± 7.8 (range: 21–51)	42.9 ± 11.5 (range: 24–55)	*t* = 1.30	0.20^b^
Daily alcohol intake (ml)^a^	497.1 ± 132.8	–		
Dependence duration (years)^a^	14.6 ± 7.2	–		
Abstinence time of hospitalization (days)^a^	34.6 ± 23.6	–		

The data are shown as the mean ± SD

^a^The data are available for 17 from 19 patients

^b^The *P* value was obtained by two-sample *t*-test

### Imaging data acquisition

Imaging data were obtained using a SignaHDx 3.0-T MR system (General Electric, Milwaukee, WI, USA). Earplugs were used to reduce scanner noise, and tight but comfortable foam padding was used to minimize head motion. DTI data were acquired using a spin-echo single-shot echo planar imaging (SE-SS-EPI) sequence with the following parameters: repetition time (TR) = 10,000 ms; echo time (TE) = 87 ms; flip angle (FA) = 90°; field of view (FOV) = 220 mm × 220 mm; acquisition matrix = 128 × 128, reconstructed to 256 × 256; slice thickness = 3 mm; gap = 0.5 mm; 36 axial slices; a voxel size of 0.9 × 0.9 × 3.5 mm^3^; 30 non-collinear diffusion gradients (b = 1000 s/mm^2^) and 1 non-diffusion-weighted images (b = 0 s/mm^2^).

### DTI data preprocessing and whole-brain fiber tracking

The software packages FMRIB Software Library (FSL, http://www.fmrib.ox.ac.uk/fsl) [39], Diffusion Toolkit (DTK, http://trackvis.org/dtk) and Pipeline for Analyzing brain Diffusion images (PANDA, http://www.nitrc.org/projects/panda) [40] were used for the DTI preprocessing steps. Specifically, the diffusion-weighted images were first registered to a reference volume (i.e., the first b0 image) by using affine transformations to minimize distortions caused by the eddy currents and head motions. After skull-stripping, we estimated the 6 independent components of the diffusion tensor from which FA, AD, RD and MD were calculated. Then a deterministic streamline tracking algorithm, i.e., Fiber Assignment by Continuous Tracking (FACT), was performed to obtain the whole-brain fiber tractography [41] with the FA threshold of 0.2 and the maximum curvature angle of 45°.

### Fiber tracking of the corpus callosum

**Fig. 1:**
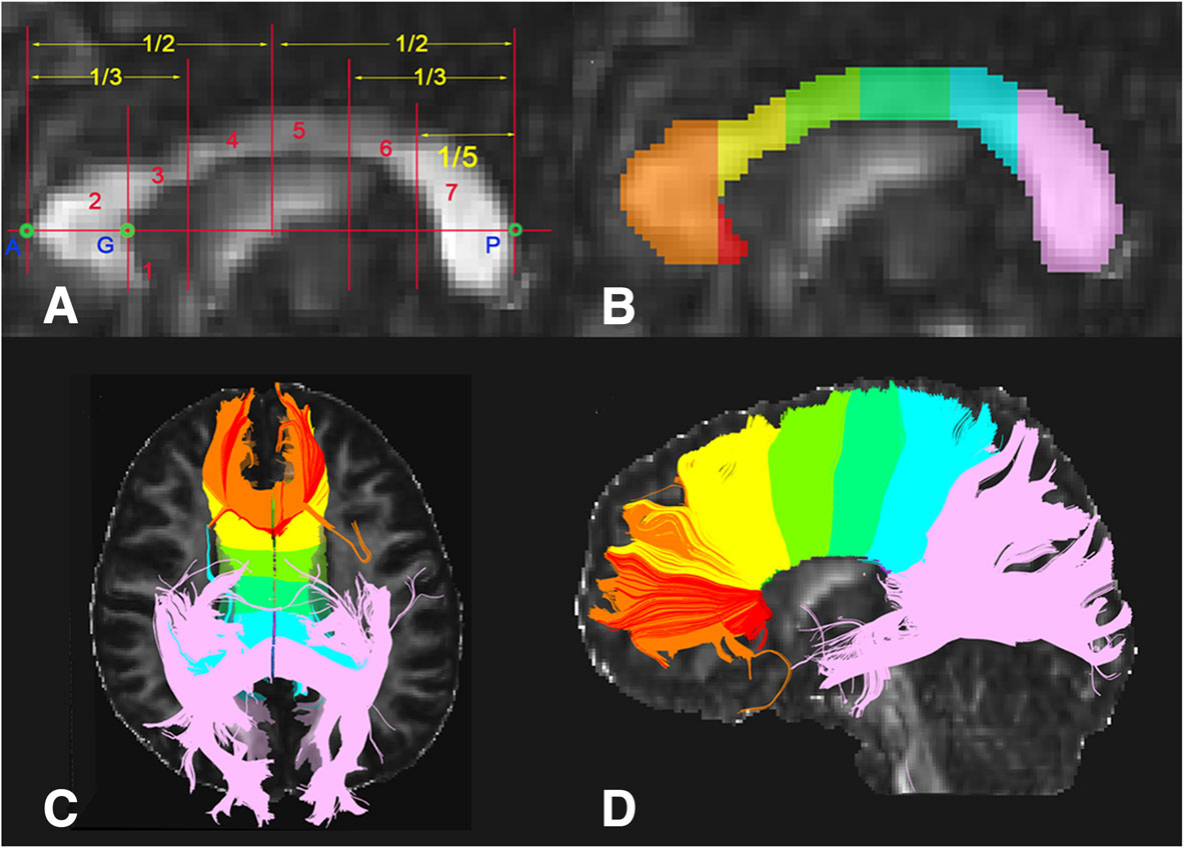
Subregions of the corpus callosum. Segmentation scheme of the corpus callosum (**a** and **b**). A and P are the anteriormost and posteriormost points, respectively. G, the anteriormost point on the inner convexity of the anterior callosum. A-P was used as the primary axis, lines perpendicular to which subdivide the corpus callosum into seven subregions. Fibers crossing through each subregion on axial and sagittal anatomical images (**c** and **d**)

The seven sub-regions of CC were defined according to a previous study [31]. The sub-regions 1–7 are rostrum (connecting the bilateral orbital prefrontal and inferior premotor areas), genu (connecting the bilateral prefrontal areas), rostral body (connecting the bilateral premotor and supplementary motor areas), anterior midbody (connecting the bilateral motor regions), posterior midbody (connecting the bilateral somaesthetic and posterior parietal regions), isthmus (connecting the bilateral superior temporal and posterior parietal areas), and splenium (connecting the bilateral occipital and inferior temporal cortical areas) (Fig. 1a). Two trained raters who were blind to subjects’ information manually divided each CC into sub-regions on the mid-sagittal section of the FA maps using TrackVis software (www.trackvis.org) (Fig. 1b). Then the whole CC and 7 sub-regions were tracked separately (Fig. 1c and d). The average FA, AD, RD and MD of the 8 fibers were extracted for each subject. The intra-class correlation coefficients (ICC) of inter-rater measures ranged from 0.85 to 1, suggesting an excellent inter-rater reliability (Additional file 1: Table S1). The mean values of the two raters’ manual measurements were calculated for subsequent statistical analyses.

### Statistical analysis

The statistical analyses were performed using SPSS 19.0 (SPSS, Inc., Chicago.IL). The inter-group differences in FA, AD, RD and MD of seven sub-regions and the whole CC were compared using two-sample *t*-tests. Associations between diffusion metrics and clinical variables in the patient group (including daily alcohol intake, dependence duration and abstinence time of hospitalization) were tested using Spearman rank order correlations. The threshold *P* < 0.01 was considered significant.

## Results

**Fig. 2:**
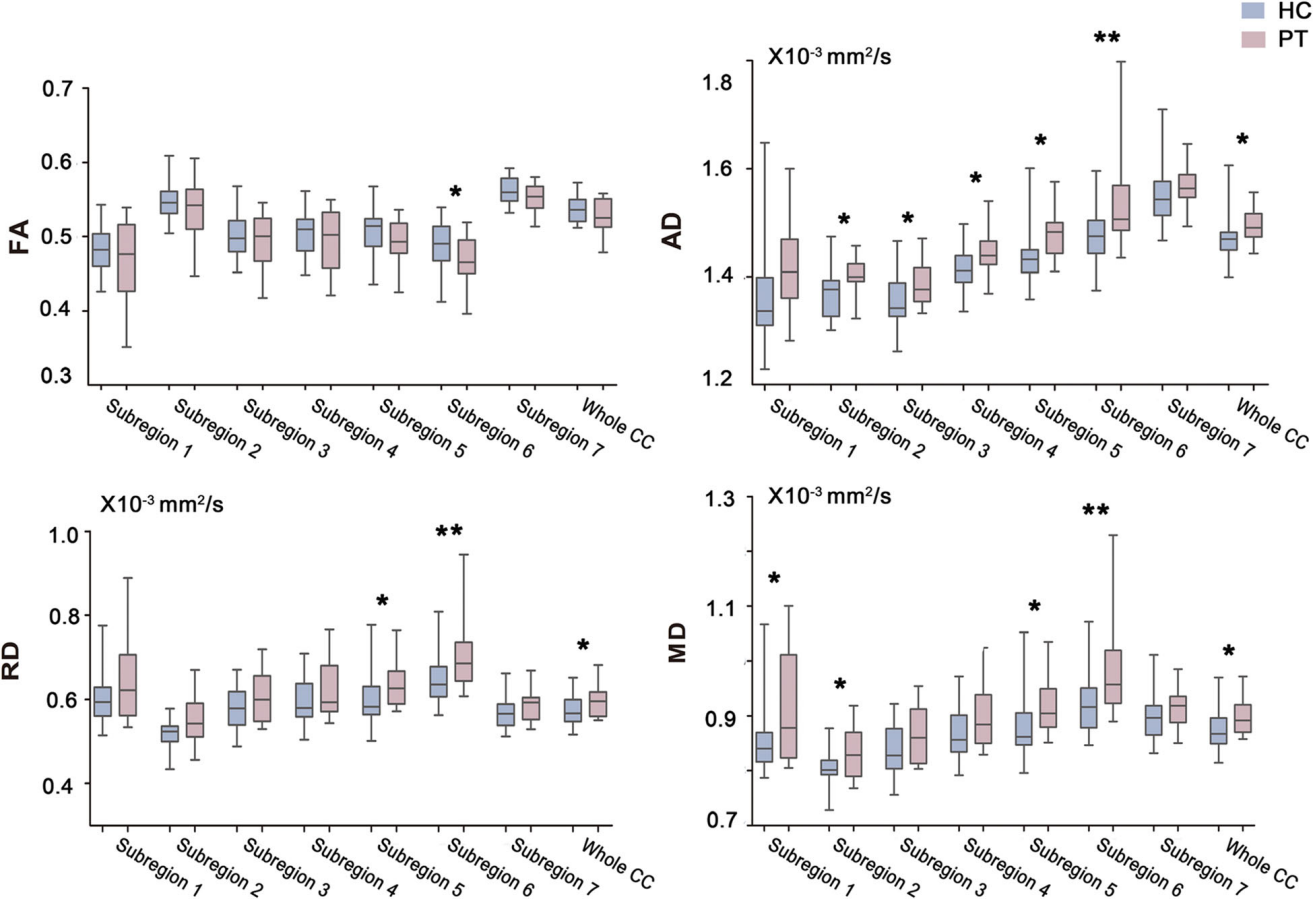
Alterations in diffusion metrics of corpus callosum subregions. The horizontal line in the middle of each box indicates the median, while the top and bottom borders mark the 75th and 25th percentiles, respectively. The whiskers above and below the box mark the maximum and minimum values. Abbreviations: CC, corpus callosum; FA, Fractional Anisotropy; AD, axial diffusivity; RD, radial diffusivity; MD, Mean diffusivity; HC, healthy controls; PT, alcohol-dependent patients

The differences in diffusion metrics of the 8 fibers between alcohol-dependent patients and healthy controls are shown in Tables 2 and 3, Additional file 1: Table S2 and Fig. 2. Compared to healthy controls, patients exhibited increased AD, RD and MD in the subregion 6 (*P* < 0.01). When using a less stringent threshold (*P* < 0.05) for illustrative purpose, altered white matter integrity was observed in the subregions 1–6 and the whole CC. To rule out the potential effect of age, we repeated the comparisons controlling for age and found that the main results were preserved (Additional file 1: Table S3).

**Table 2:** The diffusion metrics of corpus callosum subregions

Region	FA		AD		RD		MD	
	PT	HC	PT	HC	PT	HC	PT	HC
Subregion 1	0.47 ± 0.05	0.48 ± 0.03	1.41 ± 0.09	1.36 ± 0.09	0.64 ± 0.10	0.60 ± 0.06	**0.91 ± 0.09**	**0.85 ± 0.06** ^*^
Subregion 2	0.54 ± 0.04	0.55 ± 0.02	**1.40 ± 0.04**	**1.37 ± 0.04** ^*^	0.55 ± 0.06	0.52 ± 0.04	**0.83 ± 0.05**	**0.80 ± 0.04** ^*^
Subregion 3	0.49 ± 0.04	0.50 ± 0.03	**1.39 ± 0.04**	**1.35 ± 0.05** ^*^	0.60 ± 0.06	0.58 ± 0.05	0.87 ± 0.05	0.83 ± 0.05
Subregion 4	0.50 ± 0.04	0.50 ± 0.03	**1.44 ± 0.04**	**1.41 ± 0.04** ^*^	0.62 ± 0.06	0.59 ± 0.05	0.89 ± 0.05	0.87 ± 0.04
Subregion 5	0.49 ± 0.03	0.51 ± 0.03	**1.47 ± 0.04**	**1.43 ± 0.05** ^*^	**0.64 ± 0.06**	**0.60 ± 0.06** ^*^	**0.92 ± 0.05**	**0.88 ± 0.05** ^*^
Subregion 6	**0.46 ± 0.03**	**0.49 ± 0.03** ^*^	**1.53 ± 0.08**	**1.47 ± 0.05** ^**^	**0.71 ± 0.08**	**0.64 ± 0.06** ^**^	**0.98 ± 0.08**	**0.92 ± 0.05** ^**^
Subregion 7	0.55 ± 0.02	0.56 ± 0.02	1.57 ± 0.04	1.55 ± 0.05	0.59 ± 0.04	0.57 ± 0.04	0.92 ± 0.03	0.90 ± 0.04
Whole CC	0.53 ± 0.02	0.54 ± 0.02	**1.50 ± 0.03**	**1.47 ± 0.04** ^*^	**0.60 ± 0.04**	**0.57 ± 0.03** ^*^	**0.90 ± 0.04**	**0.87 ± 0.03** ^*^

** *P* < 0.01, * 0.01 < *P* < 0.05

For illustration, all values of AD, RD and MD were multiplied by 1000

Abbreviations: *CC* corpus callosum, *FA* Fractional Anisotropy, *AD* axial diffusivity; *RD* radial diffusivity, *MD* Mean diffusivity, *HC* healthy controls, *PT* alcohol-dependent patients

Entries in boldface are diffusion metrics with significant inter-group differences (*P* < 0.05)

**Table 3:** Mean inter-group differences and 95% confidence intervals in diffusion metrics of corpus callosum subregions

Region	FA			AD			RD			MD		
	Difference	Low 95% CI	High 95% CI	Difference	Low 95% CI	High 95% CI	Difference	Low 95% CI	High 95% CI	Difference	Low 95% CI	High 95% CI
Subregion 1	0.011	−0.019	0.041	−0.052	− 0.110	0.006	− 0.044	− 0.102	0.014	− 0.062	− 0.118	−0.005
Subregion 2	0.013	−0.010	0.036	−0.032	−0.058	− 0.006	−0.030	− 0.063	0.003	− 0.031	−0.058	− 0.003
Subregion 3	0.011	−0.013	0.034	−0.031	−0.060	− 0.003	−0.030	− 0.066	0.007	− 0.030	−0.062	0.002
Subregion 4	0.009	−0.014	0.031	−0.031	−0.058	− 0.005	−0.026	− 0.063	0.011	− 0.028	−0.059	0.003
Subregion 5	0.016	−0.004	0.037	−0.041	−0.071	− 0.012	−0.041	− 0.078	−0.004	− 0.041	−0.074	− 0.009
Subregion 6	0.022	0.000	0.043	−0.063	−0.110	− 0.017	−0.062	− 0.108	−0.016	− 0.062	−0.105	− 0.019
Subregion 7	0.009	−0.003	0.021	−0.021	−0.051	0.010	−0,021	− 0.045	0.003	− 0.021	−0.045	0.003
Whole CC	0.010	−0.004	0.023	−0.028	−0,053	− 0.004	−0.026	− 0.050	−0.002	− 0.027	−0.049	− 0.004

For illustration, all values of AD, RD and MD were multiplied by 1000

Abbreviations: *CC* corpus callosum, *FA* Fractional Anisotropy, *AD* axial diffusivity, *RD* radial diffusivity, *MD* Mean diffusivity, *CI* confidence interval

**Fig. 3:**
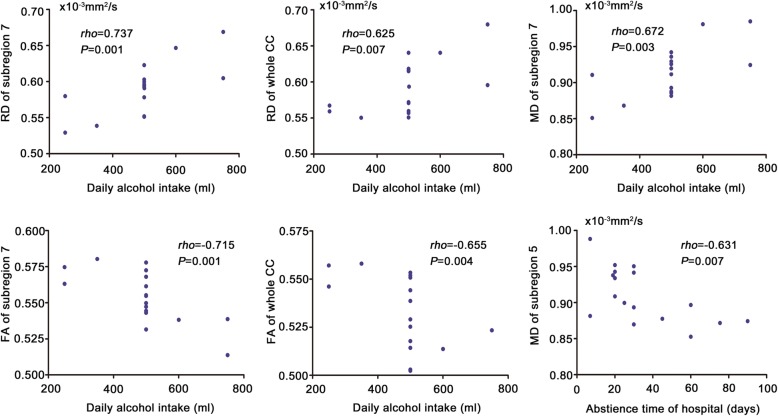
Correlations between diffusion metrics and clinical variables in the patients. Abbreviations: CC, corpus callosum; FA, Fractional Anisotropy; AD, axial diffusivity; RD, radial diffusivity; MD, Mean diffusivity; HC, healthy controls; PT, alcohol-dependent patients

In addition, we observed some significant correlations between diffusion metrics and clinical variables in the patients (Fig. 3). Specifically, daily alcohol intake was positively correlated with RD of the subregion 7 (*rho* = 0.737, *P* = 0.001) and the whole CC (*rho* = 0.625, *P* = 0.007) and MD of the subregion 7 (*rho* = 0.672, *P* = 0.003), and was negatively correlated with FA of the subregion 7 (*rho* = − 0.715, *P* = 0.001) and the whole CC (*rho* = − 0.655, *P* = 0.004). Abstinence time of hospitalization was negatively correlated with MD of the subregion 5 (*rho* = − 0.631, *P* = 0.007). We did not detect any significant correlation between dependence duration and any diffusion metrics.

## Discussion

In this study, we jointly used multiple diffusion metrics to examine the micro-structural integrity alterations of corpus callosum subregions in alcohol dependence. In comparison to healthy controls, patients exhibited 0.03–0.07 × 10^− 3^ mm^2^/s increases in the isthmus AD, RD and MD. In addition, we observed that daily alcohol intake was correlated positively with RD and MD and negatively with FA, while abstinence time of hospitalization was negatively correlated with MD in the patients.

In previous studies, alcohol dependence-related CC abnormality mainly consists of reduced volume and disrupted micro-structural integrity [15, 20–27, 42]. Pfefferbaum et al. demonstrated the microstructural degradation of the CC in alcohol-dependent patients and showed a lower FA in the genu and splenium portions [15, 42]. In another study, Liu et al. found that men with alcohol dependence showed lower FA values (0.02 to 0.03 lower) in all segments of the corpus callosum [26].

Sawyer et al. observed that compared to non-alcoholic men, alcoholic men had diminished FA (0.05 lower) in the anterior portions and body of the CC [21]. Monnig et al. reported alcohol problem severity and more frequent drinking were significant predictors of lower white matter FA in the body of CC in heavy drinkers [20]. Possible reasons for differences between the findings of this study and those in previous studies could be due to this study joint use of multiple diffusion metrics and investigation of the micro-structural integrity of the CC at the subregional level.

Among the diffusion metrics derived from DTI, FA is the most commonly used to assess white matter integrity, which measures the degree of anisotropy and ranges between 0 (fully isotropic diffusion) and 1 (fully anisotropic diffusion) [43]. However, FA changes can be driven by both parallel and perpendicular diffusivity, and thus FA is a comprehensive reflection of the water diffusion profile. Some other diffusion metrics may provide more directionally specific and complementary information [36, 37]. Specifically, AD represents the water diffusivity parallel to the axonal fibers. Altered AD may reflect axonal swelling, degeneration and deletion [44–46]. RD represents the water diffusivity perpendicular to the axonal fibers. Altered RD may reflect myelin disruption [45–48]. MD is the average of AD and RD. In this study, we found that the whole CC and its subregions of the alcohol-dependent patients exhibited alterations mainly in AD, RD and MD, which is consistent with previous studies showing that these three diffusion metrics are more sensitive than FA [36]. Therefore, a combination of multiple diffusion metrics may facilitate the detection of white matter micro-structural changes that cannot be fully captured by FA changes only. Benefitting from the advantages of subregional analyses and a combination of multiple diffusion metrics, our study may provide comprehensive insights into the integrity impairments of the CC in alcohol dependence and make a significant contribution to the emerging literature.

Alcohol dependence is often accompanied by abuse of other drugs or substances such as cigarette, cocaine and cannabis. A previous study has found an effect of chronic cigarette smoking on white matter microstructure (including the CC) in alcohol dependence [18]. Smoking and alcohol use disorder are highly comorbid, yet cigarette use has not been adequately controlled in this study because a lack of the smoking information. However, Chumin and colleagues found alcohol dependence is associated with reduced white matter integrity after controlling for the effect of cigarette smoking [49]. Further research may be needed to clarify this issue in future study. In addition, prior studies found that heavy cocaine use was associated with structural damage (0.01 to 0.05 lower in FA) in CC [17, 50]. Rigucci et al. has reported that frequent use of high-potency cannabis is associated with disturbed CC microstructural organization (0.03 × 10^− 3^ mm^2^/s higher in AD) [51].

Notably, we found that the isthmus is selectively affected among all subregions. It was characterized by increased AD, RD and MD as well as a trend towards decreased FA. This finding indicates that the underlying pathology of the isthmus may involve alterations in both axon and myelin, i.e., increased AD may be caused by axon swelling [44] and increased RD may be the result of demyelination [47, 48]. The isthmus connects the bilateral superior temporal and posterior parietal areas. The superior temporal cortex is mainly engaged in auditory processing [52], and the posterior parietal cortex (PPC) is engaged in a variety of cognitive functions including attention, working memory and learning [53–55]. Disrupted information communication between the bilateral hemispheres in these two areas may lead to the relevant clinical symptoms in alcohol dependence. Boettiger et al. found significant differences in PPC activation during decision making between individuals with alcohol dependence and healthy controls; activation in this region was also positively correlated with impulsive choice [56]. A follow-up study found that alcohol-dependent patients exhibited significantly more impulsive delayed reward discounting decision-making and had significant hyperactivity in the PPC during delayed reward discounting decisions [57]. In addition, the associations between daily alcohol intake and CC diffusion metrics are in line with prior findings indicating that drinking characteristics have an influence on brain structural and functional damages in alcoholism [12, 58, 59]. Moreover, the negative correlation between MD of subregion 5 (posterior midbody) and abstinence time of hospitalization implies that alcohol abstinence might contribute to the recovery of CC damage to some extent.

This study has several limitations. First, we did not collect information regarding education, intelligence quotient or socioeconomic status which could affect white matter integrity. The lack of these relevant data may influence our interpretation. Second, we only recruited male participants to avoid the confounding effects of gender. As gender differences in alcohol-addictive behavior have been reported [60], future studies with female subjects are of interest although female alcohol-dependent patients are scarce. Third, highly anisotropic voxels and slice gap may influence our results. DTI data with improved quality are needed in future to validate our findings. Fourth, DTI tractography has limitations to reconstruct long-distance anatomical connections, especially the inter-hemispheric CC fibers [61–63]. This disadvantage may result in an underestimation of the CC fiber number. Finally, the relatively small sample size and a lack of correction for multiple comparisons mean that our findings, although informative, remain preliminary and require replication.

## Conclusions

In summary, we jointly used multiple diffusion metrics to investigate the micro-structural integrity of the corpus callosum at the subregional level in alcohol dependence. We found the selectively affected subregion was the isthmus, where both axon and myelin alterations were present in alcohol-dependent patients.

## Additional file

Additional file 1: **Table S1.** Inter-rater reliability for FA, AD, RD and MD values in corpus callosum subregions. **Table S2.** Inter-group differences in diffusion metrics of corpus callosum subregions. **Table S3.** Inter-group differences in diffusion metrics of corpus callosum subregions after controlling age. (DOCX 28 kb)

## Abbreviations

AD: Axial diffusivity
CC: Corpus callosum
CI: Confidence interval
DTI: Diffusion tensor imaging
FA: Fractional anisotropy
HC: Healthy controls
ICC: Intra-class correlation coefficients
MD: Mean diffusivity
MRI: Magnetic resonance imaging
PPC: Posterior parietal cortex
PT: Alcohol-dependent patients
RD: Radial diffusivity
