# Supplementary material for: Selective micro-structural integrity impairment of the isthmus subregion of the corpus callosum in alcohol-dependent males
Source: BMC Psychiatry. 2019 Mar 25;19:96. doi: 10.1186/s12888-019-2079-6 (PMC6434796; doi:10.1186/s12888-019-2079-6)
Supplement: Supplementary file 1 — Table S1. Inter-rater reliability for FA, AD, RD and MD values in corpus callosum subregions. Table S2. Inter-group differences in diffusion metrics of corpus callosum subregions. Table S3. Inter-group differences in diffusion metrics of corpus callosum subregions after controlling age. (DOCX 28 kb) [file 12888_2019_2079_MOESM1_ESM.docx]

**Additional file 1: Table S1.** Inter-rater reliability for FA, AD, RD and MD values in corpus callosum subregions.

| Region |  | FA | |  | AD | |  | RD | |  | MD | |
| --- | --- | --- | --- | --- | --- | --- | --- | --- | --- | --- | --- | --- |
|  |  | *ICC* | *P* |  | *ICC* | *P* |  | *ICC* | *P* |  | *ICC* | *P* |
| Subregion 1 |  | 0.95 | <0.001 |  | 0.94 | <0.001 |  | 0.97 | <0.001 |  | 0.85 | <0.001 |
| Subregion 2 |  | 1.00 | <0.001 |  | 1.00 | <0.001 |  | 1.00 | <0.001 |  | 1.00 | <0.001 |
| Subregion 3 |  | 1.00 | <0.001 |  | 1.00 | <0.001 |  | 1.00 | <0.001 |  | 1.00 | <0.001 |
| Subregion 4 |  | 1.00 | <0.001 |  | 0.99 | <0.001 |  | 1.00 | <0.001 |  | 1.00 | <0.001 |
| Subregion 5 |  | 0.99 | <0.001 |  | 0.99 | <0.001 |  | 0.99 | <0.001 |  | 0.99 | <0.001 |
| Subregion 6 |  | 0.99 | <0.001 |  | 0.98 | <0.001 |  | 0.99 | <0.001 |  | 0.99 | <0.001 |
| Subregion 7 |  | 1.00 | <0.001 |  | 1.00 | <0.001 |  | 1.00 | <0.001 |  | 1.00 | <0.001 |
| Whole CC |  | 1.00 | <0.001 |  | 1.00 | <0.001 |  | 1.00 | <0.001 |  | 1.00 | <0.001 |

Abbreviations: CC, corpus callosum; FA, Fractional Anisotropy; AD, axial diffusivity; RD, radial diffusivity; MD, Mean diffusivity; ICC, Intra-class correlation coefficient.

**Additional file 1: Table S2**．Inter-group differences in diffusion metrics of corpus callosum subregions.

| Region |  | FA | |  | AD | |  | RD | |  | MD | |
| --- | --- | --- | --- | --- | --- | --- | --- | --- | --- | --- | --- | --- |
|  |  | *t* | *P* |  | *t* | *P* |  | *t* | *P* |  | *t* | *P* |
| Subregion 1 |  | 0.749 | 0.459 |  | -1.927 | 0.062 |  | -1.603 | 0.118 |  | **-2.138** | **0.039^*^** |
| Subregion 2 |  | 1.204 | 0.236 |  | **-2.496** | **0.017^*^** |  | -1.861 | 0.071 |  | **-2.284** | **0.028^*^** |
| Subregion 3 |  | 0.887 | 0.381 |  | **-2.112** | **0.042^*^** |  | -1.546 | 0.131 |  | -1.818 | 0.077 |
| Subregion 4 |  | 0.715 | 0.479 |  | **-2.400** | **0.022^*^** |  | -1.388 | 0.174 |  | -1.780 | 0.084 |
| Subregion 5 |  | 1.512 | 0.139 |  | **-2.653** | **0.012^*^** |  | **-2.102** | **0.043^*^** |  | **-2.412** | **0.021^*^** |
| Subregion 6 |  | **2.061** | **0.047^*^** |  | **-2.846** | **0.007^**^** |  | **-2.764** | **0.009^**^** |  | **-2.958** | **0.005^**^** |
| Subregion 7 |  | 1.532 | 0.134 |  | -1.453 | 0.155 |  | -1.769 | 0.085 |  | -1.759 | 0.087 |
| Whole CC |  | 1.446 | 0.157 |  | **-2.270** | **0.029^*^** |  | **-2.122** | **0.041^*^** |  | **-2.334** | **0.025^*^** |

** *P* < 0.01, * 0.01 < *P* < 0.05.

Abbreviations: CC, corpus callosum; FA, Fractional Anisotropy; AD, axial diffusivity; RD, radial diffusivity; MD, Mean diffusivity.

**Additional file 1: Table S3**．Inter-group differences in diffusion metrics of corpus callosum subregions after controlling age.

| Region |  | FA | |  | AD | |  | RD | |  | MD | |
| --- | --- | --- | --- | --- | --- | --- | --- | --- | --- | --- | --- | --- |
|  |  | *F* | *P* |  | *F* | *P* |  | *F* | *P* |  | *F* | *P* |
| Subregion 1 |  | 1.588 | 0.216 |  | **5.189** | **0.029^*^** |  | **4.802** | **0.035^*^** |  | **7.829** | **0.008^**^** |
| Subregion 2 |  | **4.542** | **0.040** |  | **7.582** | **0.009^**^** |  | **8.088** | **0.007^**^** |  | **10.218** | **0.003^**^** |
| Subregion 3 |  | 2.934 | 0.095 |  | **5.726** | **0.022^*^** |  | **5.639** | **0.023^*^** |  | **6.488** | **0.015^*^** |
| Subregion 4 |  | 1.675 | 0.204 |  | **5.727** | **0.022^*^** |  | 3.505 | 0.069 |  | **4.678** | **0.037^*^** |
| Subregion 5 |  | 3.446 | 0.072 |  | **7.292** | **0.010^*^** |  | **5.794** | **0.021^*^** |  | **7.050** | **0.012^*^** |
| Subregion 6 |  | **5.530** | **0.024^*^** |  | **6.892** | **0.013^*^** |  | **8.325** | **0.007^**^** |  | **8.676** | **0.006^**^** |
| Subregion 7 |  | 3.922 | 0.055 |  | 1.478 | 0.232 |  | 3.933 | 0.055 |  | 3.186 | 0.083 |
| Whole CC |  | **4.457** | **0.042^*^** |  | **5.182** | **0.029^*^** |  | **7.004** | **0.012^*^** |  | **7.227** | **0.011^*^** |

** *P* < 0.01, * 0.01 < *P* < 0.05.

Abbreviations: CC, corpus callosum; FA, Fractional Anisotropy; AD, axial diffusivity; RD, radial diffusivity; MD, Mean diffusivity.
